# Detection of events of public health importance under the international health regulations: a toolkit to improve reporting of unusual events by frontline healthcare workers

**DOI:** 10.1186/1471-2458-11-713

**Published:** 2011-09-21

**Authors:** Emily MacDonald, Preben Aavitsland, Dounia Bitar, Katrine Borgen

**Affiliations:** 1 Department of Infectious Diseases Epidemiology, Norwegian Institute of Public Health, Oslo, Norway; 2 Infectious Diseases Department, Institut de Veille Sanitaire, Paris, France

## Abstract

**Background:**

The International Health Regulations (IHR (2005)) require countries to notify WHO of any event which may constitute a public health emergency of international concern. This notification relies on reports of events occurring at the local level reaching the national public health authorities. By June 2012 WHO member states are expected to have implemented the capacity to "detect events involving disease or death above expected levels for the particular time and place" on the local level and report essential information to the appropriate level of public health authority. Our objective was to develop tools to assist European countries improve the reporting of unusual events of public health significance from frontline healthcare workers to public health authorities.

**Methods:**

We investigated obstacles and incentives to event reporting through a systematic literature review and expert consultations with national public health officials from various European countries. Multi-day expert meetings and qualitative interviews were used to gather experiences and examples of public health event reporting. Feedback on specific components of the toolkit was collected from healthcare workers and public health officials throughout the design process.

**Results:**

Evidence from 79 scientific publications, two multi-day expert meetings and seven qualitative interviews stressed the need to clarify concepts and expectations around event reporting in European countries between the frontline and public health authorities. An analytical framework based on three priority areas for improved event reporting (professional engagement, communication and infrastructure) was developed and guided the development of the various tools. We developed a toolkit adaptable to country-specific needs that includes a guidance document for IHR National Focal Points and nine tool templates targeted at clinicians and laboratory staff: five awareness campaign tools, three education and training tools, and an implementation plan. The toolkit emphasizes what to report, the reporting process and the need for follow-up, supported by real examples.

**Conclusion:**

This toolkit addresses the importance of mutual exchange of information between frontline healthcare workers and public health authorities. It may potentially increase frontline healthcare workers' awareness of their role in the detection of events of public health concern, improve communication channels and contribute to creating an enabling environment for event reporting. However, the effectiveness of the toolkit will depend on the national body responsible for dissemination and training.

## Background

By 15 June 2012, all WHO member states are expected to have implemented the surveillance and response capacities defined in Annex 1A of the International Health Regulations (2005)[1]. At the national level, member states are required to notify the WHO immediately through the National IHR Focal Point (NFP) of all events which may constitute a public health emergency of international concern [2]. At the local community and/or primary public health response level, member states are required to have implemented the capacity to "detect events involving disease or death above expected levels for the particular time and place" and report essential information to the appropriate level of public health authority. Annex 2 of the IHR (2005) provides an algorithm to assist NFPs assess which events should be reported to the WHO [1,3] and WHO has recently developed guidance material presenting 16 case scenarios to assist NFPs in these assessments at the national level [4]. However, the IHR (2005) does not specify how sub-national identification, assessment and reporting of events of potential public health significance should occur. The NFPs' ability to notify the WHO of relevant public health events is dependent on reports of events reaching the national level from the level where they originate. At the local or regional level, the threshold for deeming an event of public health significance may differ. The public health risk assessment process that occurs between healthcare workers (HCWs) and public health authorities related to an event may also need elucidation. In this context, event reporting is uniquely challenging due to a lack of definitions of what to report and prescribed timelines for when to report.

The European Centre for Disease Prevention and Control (ECDC) has proposed the "Epidemic Intelligence Framework" as a way for countries to structure national public health surveillance [5]. Epidemic intelligence includes "all activities related to early identification of potential health hazards, their verification, assessment and investigation in order to recommend public health control measures"[6]. This framework integrates indicator-based surveillance (e.g. mandatory notification of infectious diseases) and event-based surveillance into one system [5]. Event-based surveillance involves the detection, reporting, confirmation and assessment of public health events through unstructured or immediate reports [7]. An event of public health significance is not limited to infectious disease. At the international level, events of public health significance could include clusters of cases, verified epidemics or outbreaks, single cases, contamination of food, or chemical, nuclear or radiological releases [8].

The adoption of IHR (2005) requires countries to reinforce their traditional surveillance systems with a reporting system that can capture any event of public health significance [9]. Most European countries have disease reporting legislation in place that mandates the reporting of specified diseases, but the disease reporting laws differ widely throughout Europe due to the social and political context of each country. The general population's perception of health risks that require legal measures will vary according to "understandings of medical science, public appreciation of risk and public belief in the possibility of control of risk" [10]. Since 1 January 2000, member states of the EU have been obligated to report certain events involving infectious diseases through a secure web-based application to authorities in all member states and the European Commission [11]. Thus, the concept of international reporting of events is not new in Europe. However, little is known about how national authorities in the EU ensure that they are capturing relevant events from their regional or local levels. Currently, EU public health legislation is under revision with the intention to further streamline the reporting criteria in the EU with the IHR [12]. The WHO has also produced a toolkit to assist Member States incorporate the IHR (2005) into national public health legislation [13]. As of January 2010, 13 European nations had adopted new national legislation in response to the requirements of the IHR (2005)[14]. Nevertheless, while the legal framework plays an important role, widespread underreporting of legally notifiable diseases [15] indicates that legislation alone is insufficient to ensure complete reporting.

The collaborative European project REACT (**R**esponse to **E**merging infectious disease: **A**ssessment and development of **C**ore capacities and **T**ools) sought to improve coordination of infectious disease surveillance among European countries, of which one Work Package addressed local implementation of the IHR (2005). More specifically, the Norwegian Institute of Public Health's aim within the REACT project was to create tools that, in conjunction with national legislation, can assist European countries ensure unusual events observed by frontline HCWs are reported to the appropriate level of public health authority. We focused our research on identifying existing obstacles and incentives for reporting that could be helpful for designing useful tools. For the purposes of this project, frontline HCWs were defined as clinicians and microbiologists/laboratory workers.

## Methods

We used several methods including a systematic literature review, expert consultations and qualitative interviews to gather background information for the creation of the toolkit. Qualitative methods were chosen to be able to obtain a comprehensive understanding of a topic that, to our knowledge, had not previously been explored [16]. Multiple methods were used in order to increase the reliability of our findings by comparing the different results for concurrence, and, where there were discrepancies, to identify areas in need of further investigation [17].

### Systematic literature review

A literature review was conducted to find any information on obstacles and incentives to event reporting at the local and regional levels, using Ovid to search Medline and Embase. The literature review was considered an appropriate method to identify any existing resources on event reporting, help contextualize reporting practices and explain some of the existing obstacles and incentives to reporting. It was also necessary to properly frame the interview methodology and practices. Initial searches took place in March and April 2009, and were repeated in September 2009. As event reporting is a relatively new concept with sometimes inconsistent terminology, a broad exploratory search strategy was used and literature on notifiable disease reporting was also included. The search terms were initially structured in two parts to find all relevant information relating to event reporting. The first part sought to identify issues related to health event reporting, particularly at the local and regional levels, using the following key words: "report*", "underreport*", "under-report*" and "notifiable'" combined with "local", "regional", "health authority", "laboratory", "clinician", "physician", "national", "knowledge", "disease notification", "communicable disease" and "communicable disease control". The second part was structured to identify issues directly associated with national implementation of the IHR (2005), particularly in relation to identifying and reporting potential 'public health events of international concern', using the following key words: "international health regulations", "health intelligence", "epidemic intelligence" and "event based surveillance". The key words "communicable disease" and "population surveillance" were combined with the following terms: "early identification", "early warning", "early detection", "international surveillance", "event monitor*", "informal", "health event", "event surveillance", "event report*" and "event-based". No restrictions were placed on study design or language, although non-English articles were excluded through abstract screening. The initial Ovid search was limited to publications from 1990 to 2009 concerning human subjects. Literature on reporting of adverse drug reactions, occupational injuries and domestic abuse were excluded. Publications on hospital-acquired and nosocomial infection reporting were also excluded.

Additionally, a search of the internet using Google was conducted to identify further grey material, such as reporting manuals, posters and non-peer reviewed literature.

We reviewed the titles and abstracts of every record retrieved for relevancy. Articles addressing potential obstacles or incentives to event reporting in the abstract were read full text. The data obtained was subsequently synthesized using themes developed through the expert consultations. References cited in relevant articles obtained through the initial searches were checked for additional sources.

### Expert consultations

Expert consultations were planned to acquire information on the understanding and views on event reporting (in terms of needs, expectations and feasibility) from a broad base of European public health professionals. We believed themes that emerged from the literature review could be supported with primary data acquired through the expert consultations. The consultations would also elucidate differences between European countries that would need to be considered while developing the content and structure of the toolkit. Experts were invited to participate based on their knowledge of the field and experience in disease surveillance and reporting. Participants included representatives from national public health agencies, international organizations, management and legal practice with varied backgrounds, in order to collect a range of views on this specific topic. Experts were invited to participate in small group discussions and plenary sessions during two multi-day meetings, and at a later stage to provide feedback on proposed components of the toolkit.

In addition, we gathered information through standardized, open-ended interviews. The objective was to investigate obstacles and incentives to event reporting and to collect examples. Interviews provided an opportunity to evaluate existing conventions and consider recommendations on best reporting. While the group-based expert consultations allowed for discussion and interaction among the participants, the individual interviews allowed respondents time to reflect on personal experiences. The interviews were conducted using a structured guide. Purposive sampling was used to ensure representation from European countries of varying size, geographical location and political structure. Eleven national-level public health officials were invited to participate. We contacted the potential interviewees by e-mail and, if necessary, asked them to involve individuals with appropriate knowledge and experience to discuss the topic. The interview guide was piloted with two interviewees, one by telephone and one by email, from two different European countries. As both methods captured the sought information, we decided to offer interviewees the choice of responding by e-mail or by telephone. Respondents were asked for examples of successful and unsuccessful event reporting, suggestions on what could facilitate or impede successful reporting and perspectives on IHR implementation in their respective countries.

The results from the interviews were analyzed using Richie and Spencer's Framework analysis technique [18]. The interview results were coded and analysed to identify key themes and sub-themes. Themes were derived both from the aims of the study and on the basis of the interview responses. The data was then charted based on thematic content prior to interpretation. Examples were extracted to contextualize and support the findings.

Respondents' identities were kept confidential by removing any information that could jeopardize their anonymity. This included removing names of countries, country-specific tools or networks, as well as the date and time of the event. In addition, spelling and grammatical mistakes in the examples extracted from the written interviews were corrected in order to prevent identification of respondents with English as a first language versus a second language.

The research reported in this article did not require approval from an ethical committee.

## Results

### Systematic literature review

To our knowledge, no existing literature review has examined obstacles and incentives to event reporting. In total, 11,878 references were identified from which 11,355 were excluded after title screening. The abstracts were read for the remaining 523 references. Of these, 79 articles addressing obstacles or incentives to reporting in the abstract were included in the qualitative synthesis and ultimately contributed to the background information for the creation of the toolkit. Published literature on event reporting, particularly on obstacles and incentives, was very limited and the most relevant findings were related to individual reporting of notifiable disease cases.

One of the most frequently identified reasons in the literature for not reporting was lack of knowledge among clinicians of the reporting process, including not knowing what diseases are reportable and not knowing what to report [19-21]. Confusion over who is responsible for reporting between the hospital and laboratory [22] as well as confusion over whether laboratory confirmation is required prior to reporting [23] also contribute to underreporting. Silk (et al)'s review of strategies to enhance completeness of notifiable disease reporting found strengthening relationships between clinicians and other key partners to be one of the ways to encourage more complete reporting, such as by providing access to public health professionals in the case of emergencies and establishing a 24-hour toll free phone number for reporting [15]. A lack of understanding of how information acquired through reporting is used is frequently linked to underreporting. The provision of feedback is frequently recommended as a way to encourage increased reporting [24,25]. Resistance to reporting is often attributed to clinicians' lack of confidence in how reported information will be used, as is limited perceived relevance of a single clinician's contribution [23] and a perception that reporting diseases is a useless endeavour [26]. Feedback to clinicians, showing them that preventative action is being taken as a result of their notification, may be an effective way to emphasize the need for timely and complete reporting [24]. The full results of the literature review are available from the authors.

### Expert consultations

**Figure 1:**
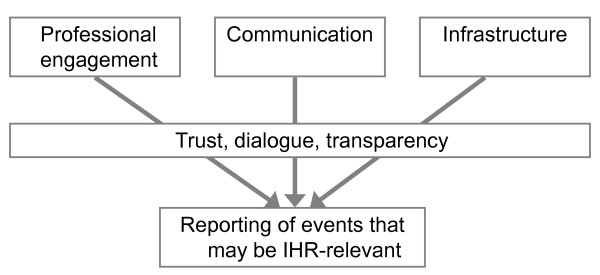
**Analytical framework used to guide design of the REACT Toolkit, developed through expert consultation**.

Two multi-day expert meetings were held, in April 2009 and June 2010, together involving more than 40 public health professionals from more than ten European countries. During the first meeting, the experts acknowledged the need among both frontline healthcare workers and public health professionals to clarify concepts and expectations around event reporting in European countries. Discussions primarily centred on the importance of creating an enabling environment for event reporting through three priority areas: 'professional engagement', 'communication' and 'infrastructure' (Figure 1). These priority areas became the foundation of the analytical framework used to guide development of the toolkit.

Professional engagement was discussed as the degree to which stakeholders are involved in the event reporting process. Professional engagement emphasises the need to increase knowledge and improve attitudes towards event reporting. This primarily refers to healthcare workers, including clinicians, laboratory staff and public health officials. It was acknowledged that although it is important to consider non-traditional sources of event reports, our primary focus was on the health sector.

Communication, or the exchange of information, both internally and across sectors or departments, was also defined as a priority area. Providing feedback to those reporting could increase trust and transparency in the exchange of information about unusual events, improve the perception of how reported information is used and demonstrate the consequences of not reporting.

Infrastructure comprises any mechanism that facilitates event reporting, including legislation, communication tools or other technological advances, like web-based reporting systems. As countries will differ in political, legal and cultural environments, it would be difficult to create one reporting tool that would be successful in all contexts. In particular, there was consensus that although a valuable component, legislation alone is unlikely to ensure complete and timely event reporting.

In total, we conducted seven qualitative interviews (one by phone, six by e-mail) with respondents from seven different European countries, including the two participants of the pilot interviews. Of the 11 individuals initially approached, we received no response from two individuals, even after sending a second request. Two individuals initially agreed to participate but did not respond after reminders. In general, the findings corroborated the results of the literature review. The interview findings also revealed the importance of authorities communicating clear expectations when providing guidance to frontline healthcare workers on reporting requirements. The effect of negative consequences to reporting, such as extra work, intrusive requests for further information, media attention, judgment, punishment or blame, was stressed as an obstacle by multiple respondents. Most of the participants emphasized the importance of open communication channels as a means to encourage reporting. The interviewees suggested providing feedback to event reports in order to place value on reporting, avoid misunderstandings and encourage cooperation. Good personal relationships or knowledge of the individuals involved in reporting was perceived to encourage reporting. Web-based reporting methods were mentioned by almost all respondents as a way to improve the timeliness and completeness of reporting. From the interviews a number of examples were extracted with the intention that these would be fictionalized and anonymously incorporated into the final toolkit.

### Toolkit for event reporting

**Figure 2:**
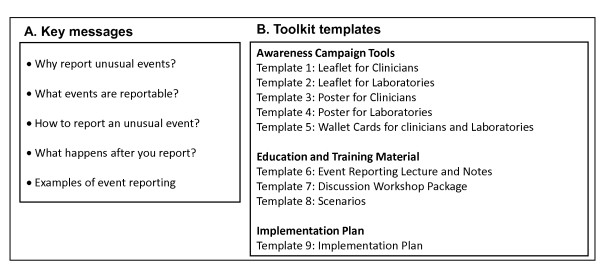
**Key messages (A) and structure (B) of the Toolkit for Local Implementation of the International Health Regulations 2005**.

Based on information collected through the literature review and expert consultations we designed a toolkit. This toolkit includes a core guidance document targeting the NFPs and nine tool templates. The guidance document contains brief background information on IHR (2005) core capacity requirements, epidemic intelligence and event-based surveillance, as well as the roles of frontline HCWs and public health authorities in event reporting. The tool templates aim to deliver five key messages (Figure 2A) and must be amended according to country specific needs prior to dissemination. The nine templates are structured in three sections: awareness campaign tools, education and training material, and an implementation plan (Figure 2B).

**Figure 3:**
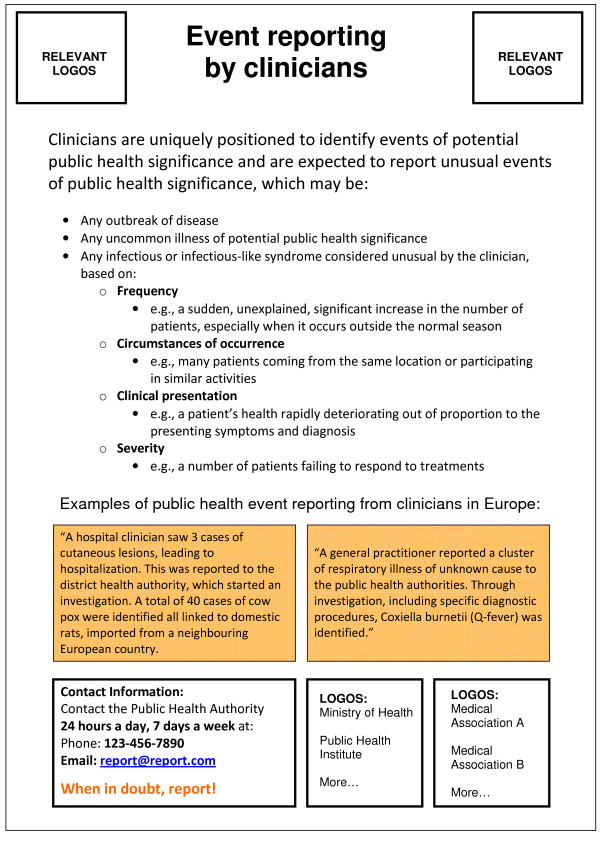
**Tool template 3: poster for clinicians**.

Templates 1 to 5 (awareness campaign tools) provide two suggested definitions of unusual events that may be seen at the frontline, one for clinicians and one for laboratories. The content is brief and supported by anonymous examples from various countries. The proposed definition for events to be reported by clinicians is: *"Any outbreak of disease*, OR *any uncommon illness of potential public health concern*, OR *any infectious or infectious-like syndrome considered unusual by the clinician, based on frequency, circumstances of occurrence, clinical presentation, or severity"*. The proposed definition of a reportable event for laboratories is: *"Any situation considered unusual related to received samples (frequency, circumstances of occurrence or clinical description) *OR *test results (unexpected number of the same species/subspecies, strain type/subtype or antimicrobial resistance pattern, or failure/uncertainty in diagnostics)"*. Figure 3 shows the poster template for clinicians included in the toolkit.

Templates 6-8 (education and training material) include a lecture with notes and references intended for an audience unfamiliar with event reporting and a discussion workshop package including a planning guide, slides and evaluation form. This package suggests a process for designing and running a one-day workshop that presents multiple short scenarios involving event reporting for discussion among frontline HCWs and public health authorities. Eleven scenarios are included in the training material, developed from real situations, and these can easily be incorporated into the lecture or discussion workshop templates.

Finally, template 9 is a suggested implementation plan with an example using information from Norway. The implementation plan presents objectives, strategies and tasks for improving event reporting from frontline HCWs by incorporating other components of the toolkit. All the tool templates are available at http://bit.ly/mkYm0V and are free to use.

## Discussion

The general objective of the REACT project was to provide evidence-driven tools, which could be applicable and acceptable throughout the EU, to promote a common European standard for the response to emerging public health threats. Our specific task within REACT was to develop concrete, practical and simple tools that could potentially be used in multiple European countries, and perhaps beyond Europe, to improve event reporting.

The proposed toolkit was created through a collaborative process, using the best available evidence. It has the potential to be useful for meeting the core capacity requirements of the IHR (2005) by focusing on frontline HCWs who are the first link in the public health reporting chain. Astute clinicians are uniquely positioned to be the first to recognize an unusual or unexpected event [27-29] and have played an important role in first identification of several high profile events of public health significance at national and international levels. Nevertheless, many health care professionals may view the request to report events as an additional inconvenience in an already busy work environment. For this reason, perhaps the most important incentive for improving event reporting is to create an enabling environment in order to foster better understanding between clinicians, laboratories and public health professionals. While the legal framework in each country plays an important role in reporting, we consider sensitization, trust and dialogue as equally important mechanisms for encouraging reporting, especially in regard to unusual events. Event reporting relies on an individual's judgment and qualitative assessment about the potentially broader implications of a public health event within a specific context. Additionally, some events of public health significance may not be identified by a country's legislation.

It is important that clinicians and laboratory workers report events that are unusual based on their own experiences and contexts, although not necessarily unusual at the national or international level. The assessment of whether an event may potentially be of international significance occurs at the national level, guided by Annex 2 of the IHR (2005) which is not intended to be used sub-nationally. In our definition of an "event" we prioritize sensitivity in order to facilitate reporting and to reduce delays, emphasizing the fact that there are no negative consequences for a potentially false signal. This might produce an increased amount of events in need of assessment by public health authorities at the local, regional or national levels, which ultimately do not require further action. A higher specificity might reduce the number of such signals, but some opportunities could be missed together with a risk of increasing the reporting delays. Another advantage of a sensitive definition is its likely contribution to an enabling environment, by creating more interpersonal interactions between the frontline HCWs and the public health authorities in charge of assessing the event and providing feedback information.

The toolkit was developed using evidence from, and with a focus on, the European context. However, NFPs from countries in other parts of the world may also find it useful. Obstacles and incentives to reporting may differ from those explored, but there may be many similarities. Additionally, although the toolkit has been developed with frontline HCWs in mind, it is possible that some of the components may be easily adapted for professionals positioned outside the health care system who could play a role in identifying events of public health concern. These may include veterinarians, or professionals working in ports, airports, transportation, agriculture, or homeland security.

There were significant challenges in designing this toolkit. Event reporting is a relatively new concept and public health authorities must clearly communicate their expectations of what events frontline HCWs should report, which is especially challenging given the inherent difficulty in defining what constitutes an unusual event. Because of the challenges of designing one single tool that can potentially be useful in multiple countries, we created a set of tool templates to be adapted with country-specific information. National public health authorities have the opportunity to choose the tools they see as appropriate for their own context and adapt them, without significant investment of time or resources. This places some responsibility on the national public health authorities to translate and prepare the documents and ensure that infrastructures are in place or under construction prior to engaging frontline HCWs in event reporting.

This work has some methodological limitations. Due to the lack of published information on event reporting, we chose to examine literature on obstacles and incentives to notifiable disease reporting. However, it is possible that the issues related to notifiable disease reporting and event reporting are less similar than assumed. The lack of available information itself indicates that event-based reporting could benefit from further research. We included articles from various countries thus addressing different reporting systems and legal frameworks which could potentially introduce bias into the synthesis of obstacles and incentives for event reporting. Limitations to the expert consultations and qualitative interviews include selection and response bias. We intentionally recruited individuals from the national or international public health levels to participate in the expert consultations. As a result, their views may not reflect specific concerns held by frontline HCWs regarding event reporting. Continued investigations on perceptions of event reporting among sub-national public health authorities and frontline HCWs could reveal unknown obstacles and incentives. In addition, only seven out of eleven invited experts participated in the qualitative interviews. Although the non-respondents may have revealed unknown obstacles and incentives, the primary goal of the interviews was to collect examples to include in the toolkit, which was satisfactorily accomplished.

## Conclusions

The toolkit described above emphasizes the importance of mutual exchange of information between frontline HCWs and public health authorities. It may potentially increase frontline HCWs' awareness of their role in detecting and reporting unusual events of potential public health concern and contribute to creating an enabling environment. Although this toolkit was designed in order to address the June 2012 IHR (2005) implementation deadline, any improvements in event reporting will have intrinsic public health benefits, in addition to simply fulfilling legal requirements. The effectiveness of this toolkit will depend on the national body responsible for dissemination, and training and implementation could benefit from collaboration with relevant medical associations. We welcome national focal points to download, modify, translate and use the toolkit available at http://bit.ly/mkYm0V.

## Competing interests

The authors declare that they have no competing interests.

## Authors' contributions

PAa, KB, EM and DB conceived of the study and participated in its design. EM and KB collected and analysed the information, designed the final toolkit and drafted the manuscript. DB and PAa participated in manuscript writing and revision. All authors read and approved the final manuscript.

## Pre-publication history

The pre-publication history for this paper can be accessed here:

http://www.biomedcentral.com/1471-2458/11/713/prepub
